# Robust and Cooperative Image-Based Visual Servoing System Using a Redundant Architecture

**DOI:** 10.3390/s111211885

**Published:** 2011-12-20

**Authors:** Nicolas Garcia-Aracil, Carlos Perez-Vidal, Jose Maria Sabater, Ricardo Morales, Francisco J. Badesa

**Affiliations:** Edificio Quorum V, Miguel Hernandez University, Avda. de la Universidad S/N, 03202 Elche, Spain; E-Mails: carlos.perez@umh.es (C.P.-V.); j.sabater@umh.es (J.M.S.); rmorales@umh.es (R.M.); fbadesa@umh.es (F.J.B.)

**Keywords:** image-based visual servoing, robotics, control

## Abstract

The reliability and robustness of image-based visual servoing systems is still unsolved by the moment. In order to address this issue, a redundant and cooperative 2D visual servoing system based on the information provided by two cameras in eye-in-hand/eye-to-hand configurations is proposed. Its control law has been defined to assure that the whole system is stable if each subsystem is stable and to allow avoiding typical problems of image-based visual servoing systems like task singularities, features extraction errors, disappearance of image features, local minima, *etc.* Experimental results with an industrial robot manipulator based on Schunk modular motors to demonstrate the stability, performance and robustness of the proposed system are presented.

## 1. Introduction

Visual servoing is a well known solution to control the position and motion of an industrial manipulator evolved in unstructured environments. The vision-based control laws can be grouped in different approaches based on the definition of the error function and the structure of the control architecture [1–3]. The two classical approaches are know as image-based visual servoing (IBVS) and position-based visual servoing (PBVS). In IBVS, the vision sensor is considered as a two-dimensional (2-D) sensor since the features are directly computed in the image space. This characteristic allows IBVS to be robust to errors in calibration and image noise. However, IBVS has some well-known drawbacks: (1) singularities in the interaction matrix or image Jacobian leading to an unstable behavior; (2) reaching local minima due to the existence of unrealizable image motions; (3) unpredictable 3D camera motion, often suboptimal cartesian and image trajectories violating some constraints of visual servoing techniques as: keeping the object in the field of view; occlusion of target due to obstacles, robot body, or self-occlusion; reaching robot joint limits and singularities in robot Jacobian; collision with obstacles or self-collision. Path planning in the image space is an elegant solution to address IBVS drawbacks. Path planning has been reported in different research papers by exploiting repulsive potential fields [4], screw-motion trajectories [5], interpolation of the collineation matrix [6], modulation of the control gains [7], polynomial parametrizations [8], and search trees in camera and joint spaces [9], parametrizing a family of admissible reference trajectories [10]. When several constraints (visibility, robot mechanical limits, *etc.*) are simultaneously considered by a path planning scheme, the camera trajectory is deviated from the optimal one. Researchers has concentrated their effort in solving some of the drawbacks of IBVS techniques, e.g., visibility constraint received a particular attention in recent past [11–14], detection and rejection of outliers based on M-estimator based statistical approach that utilizes redundancy in image features [15], an algorithm voting and consensus technique to integrate multiple visual cues to provide a robust input to the control law [16], local estimation through training of the image Jacobian which can handle non-Gaussian outliers due to illumination changes[17], robustness of 2D Visual Servoing in the presence of uncertainties in the 3D Structure [18], *etc.*

The work presented in this paper is based on our previous works [19,20]. This paper presents a robust visual servoing based on a redundant and cooperative 2D visual servoing system to solve its typical problems like task singularities, features extraction errors, disappearance of image features, *etc.* is presented. The proposed system is based on the information provided by two cameras in eye-in-hand/eye-to-hand configurations to control the 6 dof of an industrial robot manipulator.

The first approximation about the use of two cameras in eye-in-hand/eye-to-hand configurations was presented in the work of Marchand and Hager [21]. The system described in [21] use two tasks which are controlled by a camera mounted on the robot and a global camera to avoid obstacles during a 3D task. Then, in the paper reported by Flandin *et al.* [22] a system which integrates a fixed camera and a camera mounted on the robot end-effector is presented. One task is used to control the translation degrees of freedom (dof) of the robot with the fixed camera while other task is used to control the eye-in-hand camera orientation. Contrary to the two works commented before, in this paper, the proposed redundant image-based visual control can control all the 6 dof of the robot with one of the two cameras or with both at the same time in a cooperative way.

The paper is organized as follows. In Section 2, the control architecture of the cooperative image-based visual servoing system is presented. Then, some experimental results of this control scheme with an industrial robot are shown in Section 3. In the last section, the conclusions of this work are summarized.

## 2. Cooperative Eye-in-Hand/Eye-to-Hand System

Combining several sensory data is also an important issue that has been studied considering two fundamentally different approaches. In the first one, the different sensors are considered to complementary measure of the same physical phenomena. Thus, a sensory data fusion strategy is used to extract the information from multiple sensory data. The second control approach consists of selecting, among the available sensory signals, a set of pertinent data, which is then servoed. The two approaches will be referred as sensory data fusion and sensory data selection respectively.

A typical example of sensory data fusion is stereo vision. With this approach, two images provided by two different cameras are used to extract a complete Euclidean information on the observed scene. On the other hand, sensory data selection is used when all the different data no provide the same quality of information. In this case one can use data environment models in order to select the appropriate sensor and to switch control between sensors.

The approach to cooperative eye-in-hand/eye-to-hand configuration shown in this paper is clearly a case of multi sensory robot control [23]. It is considered as sensory data fusion because we assume that the sensors may observe different physical phenomena from which extracting a single fused information does not make sense. It neither pertains to sensory data selection because we consider potential situations for which it is not possible to select a set of data that would be more pertinent than others. Consequently, the proposed approach addresses a very large spectrum of potential applications, for which the sensory equipment could be extremely complex. As an improvement over previous approaches, there is no need to provide a model of the environment that would be required to design a switching or fusion strategy.

### 2.1. Controller Design

In this section the design of a redundant and cooperative image-based visual servoing controller is presented. This controller is based on the visual information provided by two cameras located respectively in eye-in-hand and eye-to-hand configurations.

The robot is supposed to be controlled by a six dimensional vector **T_E_** representing the end-effector velocity, whose components are supposed to be expressed in the end-effector frame. There are two cameras, one of them rigidly mounted on the robot end-effector (eye-in-hand configuration) and the other one observing the robot gripper (eye-to-hand configuration). Each sensor provides an *n_i_* dimensional vector signal **s_i_** where *n_i_* > 6 to be able to control the 6 dof of the robot with any one of the cameras or with the two cameras at the same time in a cooperative way. Let **s** = [**s_EIH_  s_ETH_**]*^T^* be the vector containing the signals provided by the two sensors. Using the task function formalism [24], a total error function **e** = **C**(**s** − s^*^) can be defined as:

(1) $$e=\left[\begin{array}{c} e_{\mathrm{EIH}}\\ e_{\mathrm{ETH}} \end{array}\right]=\left[\begin{array}{c} C_{\mathrm{EIH}}\\ C_{\mathrm{ETH}} \end{array}\right]\left(\left[\begin{array}{c} s_{\mathrm{EIH}}\\ s_{\mathrm{ETH}} \end{array}\right]-\left[\begin{array}{c} s_{\mathrm{EIH}}^{*}\\ s_{\mathrm{ETH}}^{*} \end{array}\right]\right)$$

where **C** = [**C_EIH_**  **C_ETH_**]*^T^* is a full rank matrix, of dimension *m* × *n_i_* (where *m* must be equal to dof to be controlled in this case *m* = 6), which allows to take into account redundant information.

An interaction matrix is attached to each sensor, such that:

(2) $$\dot{s}=\left[\begin{array}{c} {\dot{s}}_{\mathrm{EIH}}\\ {\dot{s}}_{\mathrm{ETH}} \end{array}\right]=\left[\begin{array}{cc} L_{\mathrm{EIH}} & 0\\ 0 & L_{\mathrm{ETH}} \end{array}\right]\;\left[\begin{array}{c} T_{{\mathrm{CE}}_{\mathrm{EIH}}}\\ T_{{\mathrm{CE}}_{\mathrm{ETH}}} \end{array}\right]T_{E}=L_{T}\cdot T_{\mathrm{CE}}\cdot T_{E}$$

where **T_CE_** is the transformation matrix linking sensor velocity and the end effector velocity, in the case of eye-in-hand configuration will be constant and in the other case (eye-to-hand configuration) will be variable.

**Figure 1.**
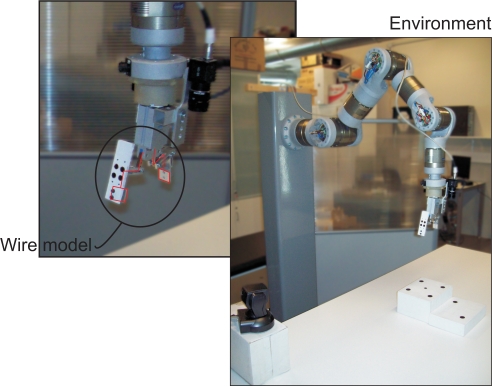
Wire model of the robot gripper with a software and environment detail.

To compute both **L_ETH_** and **T_CE_ETH__**, the mapping from the camera frame onto the robot control frame (**R, t**) must be estimated. In this paper, a model based pose estimation algorithm is used since the model of the robot gripper is a priori known [25]. To show the accuracy of the pose estimation, a wire model of the robot gripper is drawn at each iteration of the control law (Figure 1).

The time derivative of the task function (1), considering C and s* constant, is:

(3) $$\dot{e}=C\;\dot{s}=C\;L_{T}T_{\mathrm{CE}}T_{E}$$

The key in designing a task function based controller is to select a suitable constant matrix C, while ensuring that the matrix **CL_T_T_CE_T_E_** has a full rank and the system is stable. In this paper, **C** is designed as a function of the pseudo-inverse of **L_T_** and **T_CE_** with the purpose of (**CL_T_T_CE_**)^−1^ to be the identity:

(4) $$C=\left[k_{1}T_{CE_{\mathrm{EIH}}}^{-1}L_{\mathrm{EIH}}^{+}\;k_{2}T_{CE_{\mathrm{ETH}}}^{-1}L_{\mathrm{ETH}}^{+}\right]$$

where *k_i_* is a positive weighting factor such that $\sum_{i=1}^{2}k_{i}=1$.

If a task function for each sensor (where i = 1 is referred to eye-in-hand configuration and i = 2 to eye-to-hand configuration) is considered, then the task function of the entire system is a weighted sum of the task functions relative to each sensor:

(5) $$e=\sum_{i=1}^{2}k_{i}\cdot e_{i}=\sum_{i=1}^{2}k_{i}\cdot C_{i}\left(s_{i}-S_{i}^{*}\right)$$

The design of the two sensors combination simply consists of selecting the positive weights *k_i_*. This choice is both task and sensor dependent. The weights *k_i_* can be set according to the relative precision of the sensors, or more generally to balance the velocity contribution of each sensor. Also a dynamical setting of *k_i_* can be implemented.

A simple control law can be obtained by imposing the exponential convergence of the task function to zero:

(6) $$\dot{e}=-\lambda e\Rightarrow {\mathrm{CL}}_{T}T_{\mathrm{CE}}T_{E}=-\lambda e$$

where λ is a positive scalar factor which tunes the speed of convergence:

(7) $$T_{E}=-\lambda {\left({\mathrm{CL}}_{T}T_{\mathrm{CE}}\right)}^{-1}e$$

Taking into account (4), it can be demonstrated that (**CL_T_T_CE_**)^−1^ is equal to the identity:

(8) $${\left({\mathrm{CL}}_{T}T_{\mathrm{CE}}\right)}^{-1}={\left(\sum_{i=1}^{2}k_{i}T_{{\mathrm{CE}}_{i}}^{-1}L_{i}^{+}L_{i}T_{{\mathrm{CE}}_{i}}\right)}^{+}={\left(\sum_{i=1}^{2}k_{i}I_{6}\right)}^{+}=I_{6}$$

So, if C is setting to (4) and each subsystem is stable, then (**CL_T_T_CE_**)^−1^ > 0 and the task function converges to zero and, in the absence of local minima and singularities, so does the error *s* − s^*^.

Finally, substituting (4) in (7), the control law to drive back the robot to the reference position is obtained:

(9) $$T_{E}=-\lambda \left(k_{1}\cdot T_{{\mathrm{CE}}_{\mathrm{EIH}}}^{-1}L_{\mathrm{EIH}}^{+}e_{\mathrm{EIH}}+k_{2}\cdot T_{{\mathrm{CE}}_{\mathrm{ETH}}}^{-1}L_{\mathrm{ETH}}^{+}e_{\mathrm{ETH}}\right)$$

**Figure 2.**
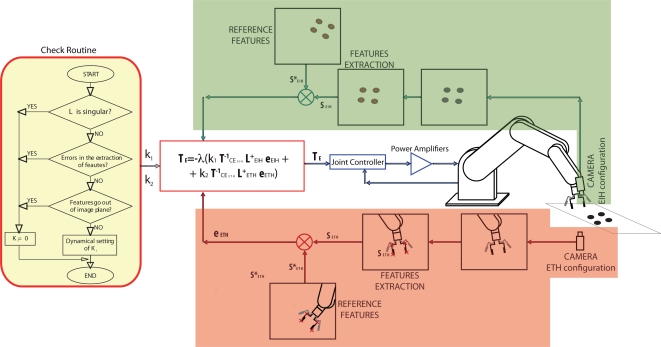
General architecture of the proposed controller.

**Figure 3.**
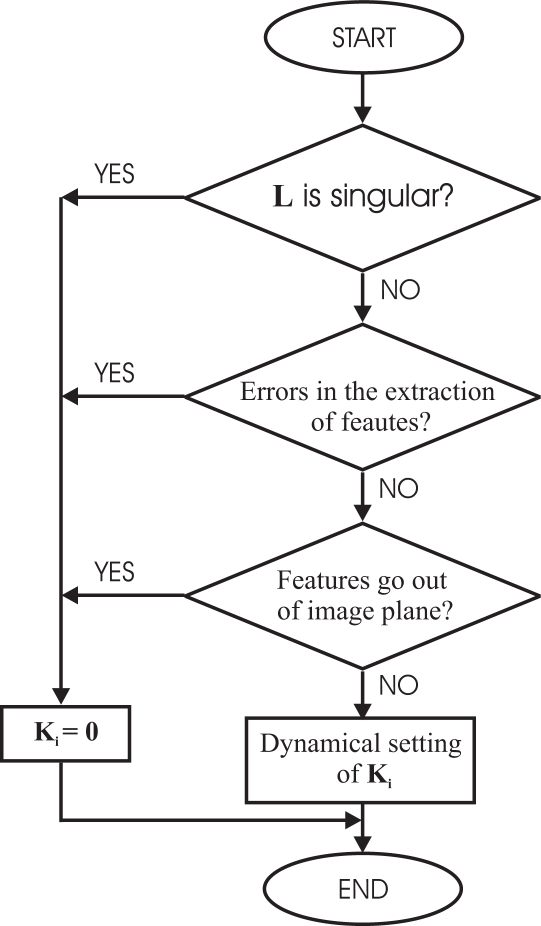
Flow chart of the routine used to detect the potential problems of image-based visual servoing systems.

In Figure 2, a control scheme of the general architecture proposed by the authors can be seen. To implement it, a software function (Check routine) to give the corresponding values to *k*_1_ and *k*_2_ is used. This Check routine is shown in Figure 3 as flowchart.

### 2.2. Controller Implementation

It is obvious that the performance of the proposed system depends on the selection of the weights *k_i_*. Before giving the corresponding value to *k_i_* some rules have been taken into account to avoid typical problems of image-based visual servoing approaches like task singularities, features extraction errors, disappearance of features from the image plane, *etc*. To do this, a checking routine is executed and if one of the problems described before are produced, the corresponding value of *k_i_* will set to zero. In Figure 3, the flow chart of the checking routine can be seen. Obviously, the system fails if the problems happens in both configurations at the same time.

In Figure 3, the dynamical setting of *k_i_* box represents a function to give values to *k_i_* depending on some predefined criteria. In this paper, *k_i_* is computed in each sample time by the following function that depends on the relative image error:

(10) $$k_{1}=\frac{e_{{\mathrm{rel}}_{\mathrm{EIH}}}}{e_{{\mathrm{rel}}_{\mathrm{EIH}}}+e_{{\mathrm{rel}}_{\mathrm{ETH}}}}\;\;\;\;\;\;\;\;\;\;\;\;\;\;k_{2}=\frac{e_{{\mathrm{rel}}_{\mathrm{ETH}}}}{e_{{\mathrm{rel}}_{\mathrm{EIH}}}+e_{{\mathrm{rel}}_{\mathrm{ETH}}}}$$

where:

(11) $$e_{{\mathrm{rel}}_{i}}=\frac{s_{i}\left(t\right)-s_{i}^{*}}{s_{i}\left(0\right)-s_{i}^{*}}$$

Note that **e_rel_EIH__** is computed when *i* = 1 and then is normalized through dividing it by the number of image features. In the same way, **e_rel_ETH__** is obtained.

The key idea of using this function is that the control contribution due to one of the cameras has more effect when its image features are far from their reference position. With this formulation of variable *k_i_*, the local minima problems are avoided since the change in the weights *k_i_* will bring the system away from it. So we can assure that **e** = 0 *if and only if* **e***_i_* = 0 ∀ *i*.

## 3. Experimental Results

Experimental results has been carried out using a 7 axis redundant robot manipulator (only 6 of its 7 dof have been considered) based on Schunk modular motors. This robot has been designed and manufactured especially to perform visual servoing tasks and has a maximum allowable load of 10 Kg.

This robot (shown in Figure 1) is mounted using 7 PRL modules (two PRL-120, two PRL-100, two PRL-80 an one PRL-60) and links made of aeronautical aluminum (manufactured using a 5-axis milling machine). PRL modules are connected by a CAN-Open bus to a PCI CAN controller (ESD-electronics).

**Figure 4.**
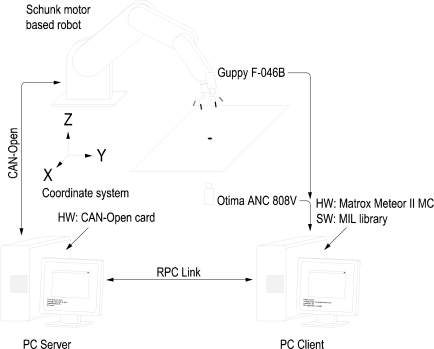
Experimental setup. The robot is controlled with a PC (server) connected with a visual processing computer (client) via RPC.

**Figure 5.**
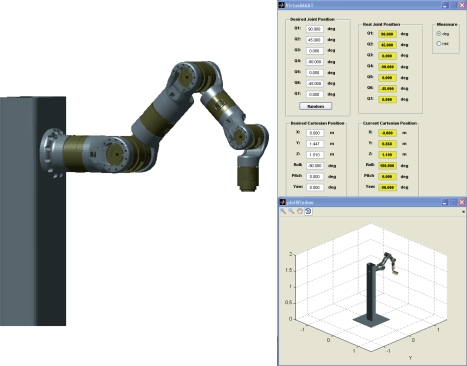
Images of the simulation environment implemented using Matlab and Simulink.

The experimental setup used in this work also includes a firewire camera (model Guppy F-046B, monochromatic, resolution of 780 × 582, 49 fps, Allied Vision Technologies) rigidly mounted in the robot end effector, a camera (manufactured by Otima, model ANC 808V Wired Type) observing the robot gripper, some experimental objects and a computer with a Matrox Meteor II MC vision board and other computer with a CAN-Open card to control the Schunk motor based robot. An RPC link between the robot controller and the computer with the vision board for synchronization tasks and data interchange has been implemented. The whole experimental setup can be seen in Figure 4. Moreover, a simulation environment has been implemented using Matlab and Simulink to test the control algorithms before to corroborate the simulation results in the experimental platform (shown in Figure 5). In the simulation environment, the robot dynamics and kinematics, camera models, errors in the extraction of image features, *etc.* have been considered in order to carry out simulations as close to real experimental environment as possible.

**Figure 6.**
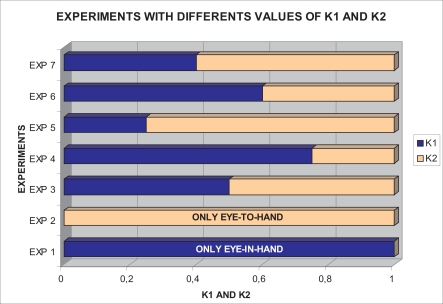
Experiments with different values of *k*_1_ and *k*_2_.

**Figure 7.**
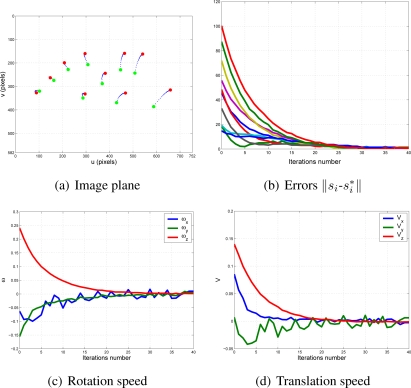
Results with *k*_1_ = 1, *k*_2_ = 0. Only the results of the camera in eye-in-hand configuration are shown. The translation and rotation speeds are measured in $\frac{m}{s}$ and $\frac{\deg}{s}$.

**Figure 8.**
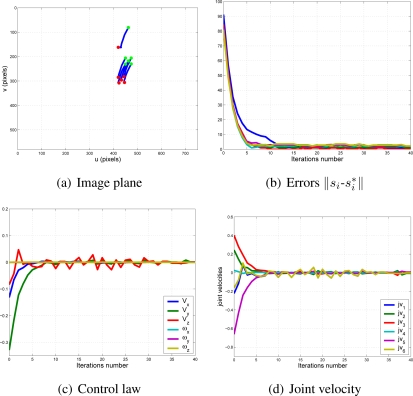
Results with *k*_1_ = 0, *k*_2_ = 1. Only the results of the camera in eye-to-hand configuration are shown. The translation and rotation speeds are measured in $\frac{m}{s}$ and $\frac{\deg}{s}$.

With this experimental setup, exhaustive number of experiments have been made with different constant weights during the control task (see Figure 6). In Figures 7 and 8, the results with *k*_1_ = 1, *k*_2_ = 0 (only the camera in eye-in-hand configuration is used) and *k*_1_ = 0, *k*_2_ = 1 (only the camera in eye-to-hand configuration is used) are presented. In these experiments, we could verify that each system is stable and the error tends to zero except the noise of feature extraction.

**Figure 9.**
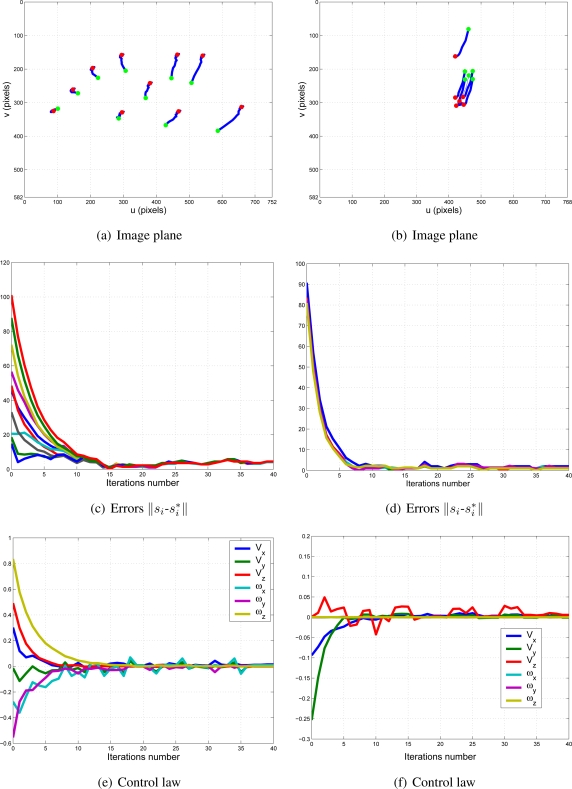
Results with *k*_1_ = 0.5, *k*_2_ = 0.5. Figures (a,c,e) are the results of the camera in eye-in-hand configuration and figures (b,d,f) are the same for the camera in eye-to-hand configuration. The translation and rotation speeds are measured in $\frac{m}{s}$ and $\frac{\deg}{s}$.

**Figure 10.**
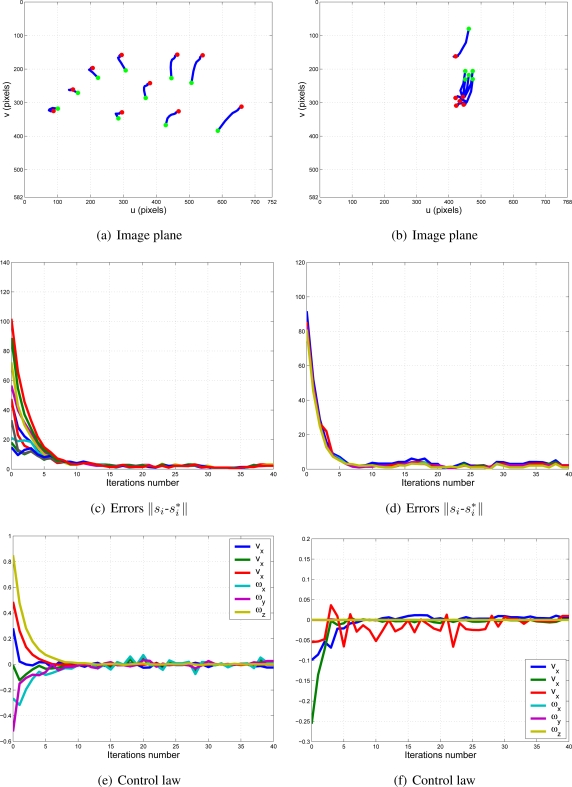
Results with *k*_1_ = 0.75, *k*_2_ = 0.25. Figures (a,c,e) are the results of the camera in eye-in-hand configuration and figures (b,d,f) are the same for the camera in eye-to-hand configuration. The translation and rotation speeds are measured in $\frac{m}{s}$ and $\frac{\deg}{s}$.

To adjust the value of *k*_1_ and *k*_2_, several experiments have been carried out. In Figures 9 and 10, the structure of the whole system is used since *k*_1_ ≠ 0 and *k*_2_ ≠ 0. In short, Figure 9 shows the obtained results with a constant weight of (*k*_1_ = 0.5 and *k*_2_ = 0.5). It means that both cameras contribute with 50% to the global control signals. Figure 10 shows the results with a constant weight of (*k*_1_ = 0.75 and *k*_2_ = 0.25). Taking a look carefully to the Figures 7–10, and the results of all the experiments carried out, we can realize that the system is stable and independent to the values of *k_i_*. These experiments corroborates the stability analysis presented at the end of Section 2. Assuring that each system is stable, the cooperative control system allows us to modify the magnitude of *k_i_* without risk of making the system unstable.

**Figure 11.**
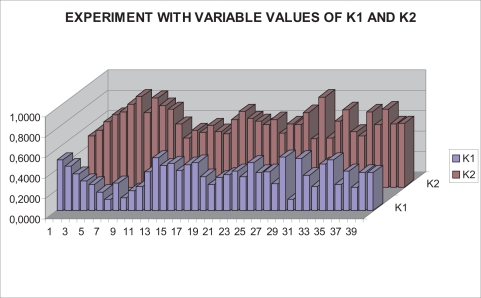
Experiments with variable values of *k*_1_ and *k*_2_.

**Figure 12.**
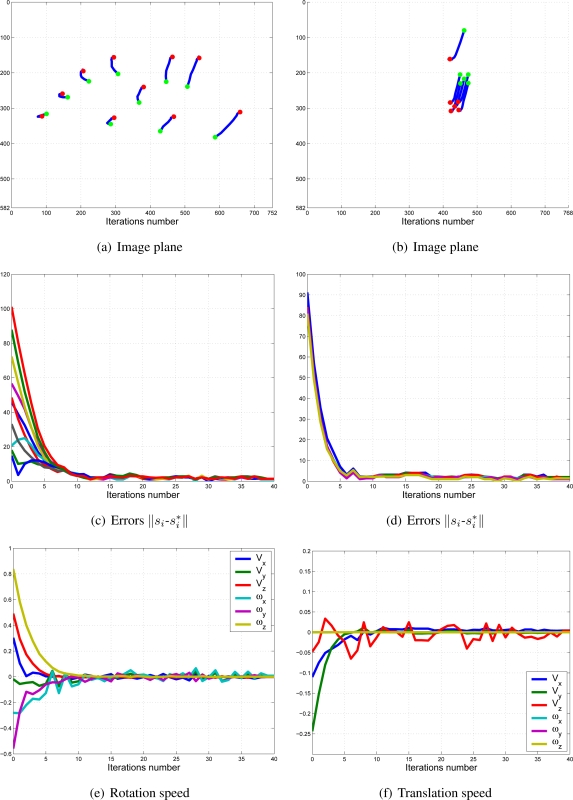
Results with variable values of *k*_1_ and *k*_2_. Figures (a,c,e) are the results of the camera in eye-in-hand configuration and figures (b,d,f) are the same for the camera in eye-to-hand configuration. The translation and rotation speeds are measured in $\frac{m}{s}$ and $\frac{\deg}{s}$.

In this paper, the dynamical setting of *k_i_* (10) is used to carry out a huge number of experiments. In Figure 11, the values of *k*_1_ and *k*_2_ during the control task in one of the experiments can be seen. In Figure 12, the results of using a variable value of the weights are shown. Observing them, we can realize that the system is stable and the error tends to zero except the noise of feature extraction.

**Figure 13.**
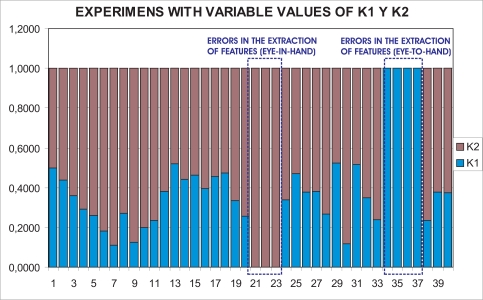
Values of *k*_1_ and *k*_2_ with forced error in the extraction of features.

**Figure 14.**
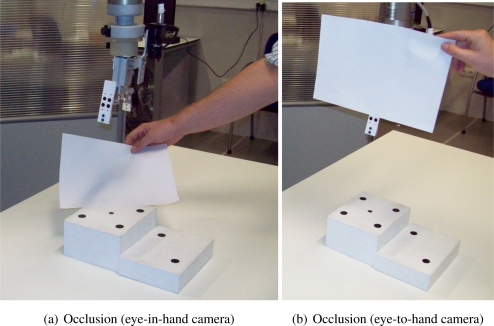
Images of the errors which are produced deliberately by the occlusion of features during the control task.

To show the performance of the proposed system with typical problems of image-based visual servoing approaches like task singularities, features extraction errors, disappearance of features from the image plane, many experiments have been carried out. The results of a simple experiment where features extraction errors are produced deliberately are shown in Figure 13. Observing Figures 13 and 14, we can see that:

- Iterations 20–22: an error in the extraction of features (eye-in-hand configuration) is produced deliberately (Figure 14(a)). This error is detected by the checking routine (Section 2.2) and *k*_1_ is set to zero.
- Iterations 33–36: an error in the extraction of features (eye-to-hand configuration) is produced deliberately (Figure 14(b)). This error is detected by the checking routine (Section 2.2) and *k*_2_ is set to zero.

In spite of these forced errors, the system is stable and the robot reaches its reference position accurately.

## 4. Conclusions

The redundant and cooperative visual servoing system proposed in this paper has been designed to make more robust the classical imaged based visual servoing systems. In all experimental results, the positioning accuracy of the architecture presented in this paper is better than the classical one and also problems like local minima, task singularities and features extraction errors are avoided. Moreover, the proposed architecture allows also to use several kinds of sensors like cameras, force sensors, *etc.* without excessive difficulty.

As future work, new functions to give values of *k_i_* are been analyzed to obtain an online method of parameter adjustment.
